# Synergistic protective effect of *Betavulgaris* with meso-2,3-dimercaptosuccinic acid against lead-induced neurotoxicity in male rats

**DOI:** 10.1038/s41598-020-80669-4

**Published:** 2021-01-08

**Authors:** Nadia Z. Shaban, Sara E. Abd El-Kader, Fayed A. K. Mogahed, Mohamed A. L. El-Kersh, Noha H. Habashy

**Affiliations:** 1 Biochemistry Department, Faculty of Science, Alexandria University, Alexandria, 21511 Egypt; 2 Department of Nucleic Acid Research, Genetic Engineering and Biotechnology Research Institute, City of Scientific Research and Technological Applications (SRTA-City), New Borg El-Arab, Alexandria, 21934 Egypt

**Keywords:** Biochemistry, Drug discovery, Neuroscience, Medical research, Neurology

## Abstract

Lead (Pb) toxicity is one of the most prevalent causes of human neurotoxicity. The available chelator drugs used now have many adverse effects. So, in this study, the protective role of *Beta* *vulgaris* juice (BVJ) on rat neurotoxicity induced by Pb was evaluated and the results were compared with the results of dimercaptosuccinic acid (DMSA, as used drug). Additionally, the synergistic effect of BVJ and DMSA against Pb-induced neurotoxicity was assessed. The study focused on the determination of the antioxidant, anti-inflammatory, and neurological potential of BVJ (alone, and with DMSA) towards lead-induced neurotoxicity. Also, the characterization of BVJ was studied. The results showed that BVJ contains considerable quantities of polyphenols, triterpenoids, and betalains which play an important role as antioxidants and anti-inflammatory. BVJ exhibited a protective effect against neurotoxicity via the reduction of Pb levels in blood and brain. Moreover, BVJ decreased the oxidative stress, inflammation, and cell death induced by Pb. Also, BVJ regulated the activities of acetylcholine esterase and monoamine oxidase-A which changed by Pb toxicity. BVJ and DMSA combination displayed a synergistic antineurotoxic effect (combination index ˂ 1). These results were in harmony with brain histopathology. Conclusion: BVJ has a powerful efficacy in the protection from brain toxicity via diminishing Pb in the brain and blood circulation, resulting in the prevention of the oxidative and inflammatory stress. Treatment with BVJ in combination with DMSA revealed a synergistic effect in the reduction of neurotoxicity induced by Pb. Also, the antioxidant and anti-inflammatory effects of the BVJ lead to the improvement of DMSA therapy.

## Introduction

Heavy metal are classified into essential and nonessential metals^1^. Essential metals including manganese, cobalt, iron, and zinc, are present in trace amount in the body and have pivotal roles as cofactors, coenzymes, and/or mediators. In contrast, if human or any organism exposed to a high amount of these metals, they become poisonous^1,2^. While, environmental contamination and exposure to numerous nonessential heavy metals which have no known benefit for human, animal and aquatic organisms physiology^3–5^, such as lead (Pb), arsenic cadmium, and mercury, is a dangerous growing universal problem^4,5^, especially in some developing countries including Egypt^6^.

Pb is the oldest toxic heavy metal that exists in several occupational and environmental sources as soils, rocks, water, and the aquatic environment^4,7,8^. Pb can introduce the body via three main routes: inhalation, ingestion, and dermal contact and subsequently distributed between red blood cells (RBCs) and soft and mineral tissues^16^. Inorganic Pb is not metabolized by living systems, finally leading to its accumulation up to toxic levels^12^. Pb toxicity poses risks to the very young, compromising development due to early life exposure, leading to lifelong physical, behavioral, and intellectual impairments^9,10^. Individuals with an elevated body load of heavy metals are more procumbent to diseases such as neurotoxicity, diabetes, cardiovascular diseases, infertility, risk of renal damage, and cancer^9,13^. Because, Pb and its compounds interfere with the functions of different organs and systems such as, the nervous system, the hematopoietic system, liver, and kidney^9^. Pb intoxication may cause blood vessels and tissue inflammation, making more calcium to be dragged to the area as a buffer and resulting in stiffening of the arterial walls with progressive blockage of the arteries and osteoporosis^14,15^. Since calcium is a critical ion in neuronal function, including cell growth and differentiation, neurotransmitter release, and intracellular biochemical cascades^17^. Thus, it has been showed that the neurotoxicity induced by Pb is arised because of its ability to replace some essential metals in the brain especially calcium ion (Ca^2+^)^18,19^. This will disrupt various biological processes including ionic conduction, metal transport system, cell adhesion, inter- and intracellular signaling, apoptosis, and others^16,20^. Moreover, Pb has high affinity to important functional groups including carboxyl, amino, and especially sulfhydryl groups and thus it can interfere with several biomolecules and enzymes^8^. These includethe molecules that are involved in the antioxidant defense system such as reduced glutathione (GSH), glutathione peroxidase (GPx), catalase, and superoxide dismutase (SOD). As a result, the antioxidant system hemostasis will be disrupted leading to the generation of reactive oxygen species (ROS) and induction of oxidative stress (OS)^4,11^, resulting in several neurodegenerative diseases such as Alzheimer, Parkinson, and Schizophrenia^19^.

Acute and chronic Pb intoxications can be effectively treated in many cases by using chelation therapies^21^ such as Edetate calcium disodium, Dimercaprol, 2,3-dimercaptopropanol, 2,3-dimercapto-1-propane sulfonic acid, and meso-2,3-dimercaptosuccinic acid (DMSA). Chelating agents convert Pb ions with different mechanisms into a chemically and biochemically inert form that can be excreted in urine^22^. For example, the calcium in Edetate calcium disodium can be displaced by divalent and trivalent metals, especially lead, to form stable soluble complexes which can then be excreted in urine^8,21^. Otherwise, lead and some other heavy metals act by chemically reacting with neighboring thiol residues on metabolic enzymes, creating a chelate complex that inhibits the affected enzyme's activity. Dimercaprol, DMSA or any chelating agentes containing thiol group, competes with the thiol groups for binding the Pb ion which is then excreted in the urine^22^.

Unfortunately, most of these chelators have drawbacks and cause renal failures, tetany, hypocalcemia, hypotension, bone marrow depression, prolonged bleeding time, convulsions, as well as respiratory arrest^23^. The DMSA is a less toxic and most effective chelating drugin decreasing Pb levels of blood and tissues via the binding Pb with its sulfhydryl groups, forming complex which excreted in urine^24^. In addition, it has a higher LD50 (more than 3 g/kg in mice and rats) as compared to other chelators^21^. However, DMSA cannot cross the cell membrane and the blood–brain barrier^25^. Also, it may cause gastrointestinal anxiety, skin irritation, mild neutropenia, and transient elevation in liver enzymes. Furthermore, it chelates essential metals such as calcium and iron^26,27^.

The previous studies revealed that the supplementation of antioxidants and mineral-rich nutrients with the chelating agents has confirmed to be a better treatment style^26–28^. Numerous edible plants are known to possess antioxidant properties, as they are rich in phytoantioxidants, namely phenolic compounds, flavonoids, etc.^1,26,29^. These natural antioxidants may improve metal mobilization from the body, reduce the dose of potential toxic chelators, and restrict the redistribution of toxic metal from one organ to another^30^.

Beetroot (*Beta vulgaris* L., BV) is a high-nutrient vegetable that belongs to the Chenopodiaceae family. It is usually consumed as a food and used as a natural food colorant (E162) and a medicinal plant in Europe. The BV is a great source of antioxidant compounds and it is a nitrate-rich plant contains the water-soluble nitrogenous pigments named betalains “red betacyanins and the yellow betaxanthins"^31^. Also, it is a good source of polyphenols, minerals, and vitamins. Recent studies have proved that beet root ingestion shows beneficial physiological effects and improves several pathologies, such as hypertension, atherosclerosis, type 2 diabetes, cardiovascular diseases, hepatotoxicity and dementia^26,31^. Therefore, in this study, we used BVJ alone and with DMSA in the treatment of neurotoxicity induced by Pb in male rats. Where BVJ may reduce the OS and apoptosis induced by Pb, maintain the levels of the essential minerals in the rat body, may improve DMSA therapy, and may enhance Pb elimination. Likewise, BVJ may ameliorate the hemoglobin level, resulting in the improvement of the circulatory system and the cerebrovascular blood flow, which in turn prevent cognitive disorders. The study focused, for the first time, on the determination of the possible synergistic antineurotoxicity, antioxidant, antiapoptotic and anti-inflammatory actions of the treatment with (BVJ and DMSA together) against Pb toxicity. Moreover, the chemical composition and antioxidant potential of the BVJ were evaluated for a better explanation of its probable antineurotoxicity role.

## Results

### Characterization of BVJ

**Figure 1:**
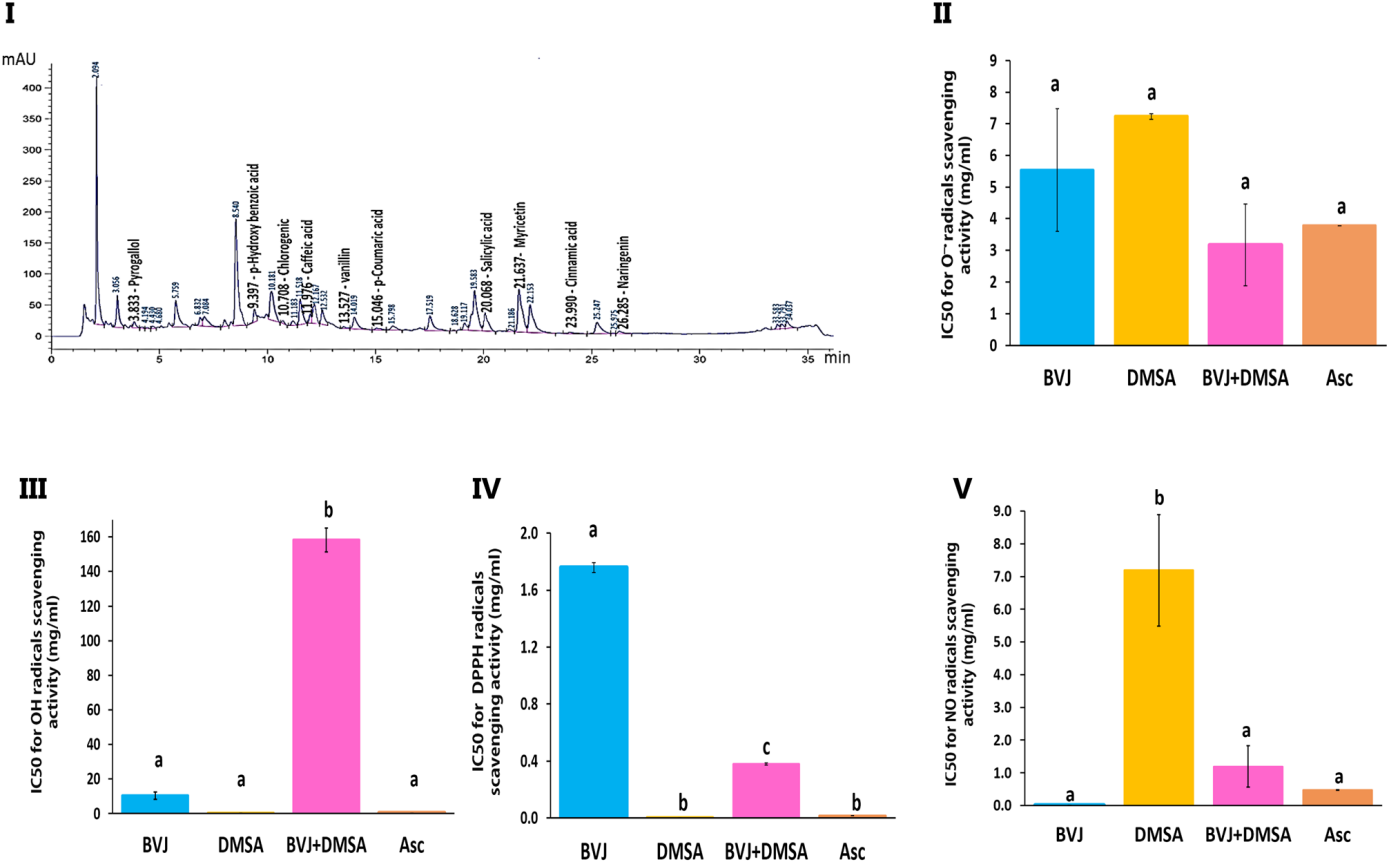
HPLC analysis and in vitro antioxidant activities of *Beta vulgaris* juice (BVJ), dimercaptosuccinic acid (DMSA), and their combination (BVJ + DMSA) compared to ascorbic acid (Asc). Where (**I**): HPLC analysis of BVJ, (**II**): superoxide radical (O·‾) scavenging activitiy, (**III**): hydroxyl radical (OH^−**۰**^) scavenging activity, (**IV)**: α,α-diphenyl-β-picrylhydrazyl (DPPH) scavenging activity, and (**V**) nitric oxide (NO), scavenging activity. The results are shown as mean ± SE (n = 3). Different letters for the same parameter are significantly different at *p* < 0.05.

The present results showed that BVJ contains various phytochemical constituents with different concentrations as shown in (Table 1). Where, the phenolics, betacyanins, betalains, betaxanthins, flavonoids, flavonols, and triterpenoids are present in a large amounts. Additionally, the high-performance liquid chromatography (HPLC) analysis of BVJ revealed the presence of many phenolic and flavonoid compounds identified against known phenolic standards by comparing their corresponding retention times (Fig. 1I and Table 1). Where, Myricetin, Quercetin, Naringenin, Pyrogallol, Caffeic acid and Chlorogenic acid exist in great amounts while Catechol and Kaempferol are not present (Table 1).

**Table 1:** Phytochemical content and phenolics HPLC analysis of *Beta vulgaris* juice (BVJ).

Phytochemical constituents	Content
Total betalains (mg g^−1^ BVJ)	5.73 ± 0.04
Betacyanins (mg g^−1^ BVJ)	3.68 ± 0.07
Betaxanthins (mg g^−1^ BVJ)	2.04 ± 0.05
Triterpenoids (µg UA eq g^−1^ BVJ)	25.16 ± 0.722
Flavonols (µg QR Eq g^−1^ BVJ)	606.40 ± 0.00
Total phenolics (mg GA Eq. g^−1^ BVJ)	17.26 ± 0.825
Total flavonoids (mg QR Eq. g^−1^ BVJ)	1.47 ± 0.00
**HPLC analysis of phenolic compounds (µg g**^**−1**^** BVJ)**	
Pyrogallol	44.095 ± 0.739
p-Hydroxy benzoic acid	191.900 ± 1.572
Chlorogenic acid	10.860 ± 0.548
Caffeic acid	20.915 ± 0.680
Vanillin	8.193 ± 0.380
p-Coumaric acid	6.878 ± 0.201
Salicylic acid	476.725 ± 2.783
Myricetin	3979.998 ± 110.604
Cinnamic acid	3.276 ± 0.146
Quercetin	881.732 ± 4.585
Naringenin	212.253 ± 1.582
Catechol	ND
Kaempferol	ND

The results are presented as mean ± SE (n = 3). *QR* quercetin, *UA* ursolic acid,* GA* gallic acid, *Eq* equivalent, *ND* not detected.

### In vitro antioxidant properties of BVJ alone and with DMSA

The results demonstrated the potent scavenging ability of BVJ to α,α-diphenyl-β-picrylhydrazyl (DPPH), Nitric oxide (NO), superoxide anion (O_2_^–**۰**^), and hydroxyl (OH^−**۰**^) radicals (Fig. 1II–V). The scavenging ability of BVJ to NO radical was significantly (p ˂ 0.05) superior (lower IC50 value) than that of ascorbic acid (Asc) but was significantly inferior (higher IC50 value) than those of DPPH and OH^‾**۰**^radicals. Additionally, the same potency was observed to O_2_^–**۰**^ radical. As well, DMSA had scavenging power similar to Asc for DPPH, O_2_^–**۰**^, and OH^‾**۰**^, while its ability to scavenge NO was significantly (*p *˂ 0.05) lower than it. Concerning the synergistic analysis, the combination of BVJ and DMSA (BVJ + DMSA) showed a synergistic (combination index "CI" ˂ 1) scavenging effect for DPPH, NO, and O_2_^–**۰**^. In contrast, it exhibited an antagonistic (CI* >* 1) scavenging effect on OH^‾·^ (Table 2). The scavenging power of BVJ and DMSA in combination for the NO and O_2_^−**۰**^ radicals was similar to Asc. While The scavenging power of this combination for DPPH and OH^‾^ radicals was lower than the Asc.

**Table 2:** The changes in the hematological parameters and levels of serum electrolytes in all the studied groups.

Groups	C	Pb	BVJ + Pb	Pb + DMSA	BVJ + Pb + DMSA	BVJ
Hb (g/dL)	14.03 ± 0.58^ad^	9.26 ± 0.58^b^	13.90 ± 1.73^ad^	10.84 ± 0.54^bc^	12.30 ± 0.31^ cd^	15.77 ± 0.42^a^
RBCs (miL/Cmm)	6.23 ± 0.34^ac^	4.31 ± 0.26^b^	6.85 ± 0.72^a^	5.57 ± 0.28^c^	6.21 ± 0.26^ac^	7.11 ± 0.31^a^
HCT (%)	36.51 ± 0.82^ac^	26.64 ± 2.23^b^	35.54 ± 3.93^ac^	30.78 ± 1.18^bc^	32.88 ± 0.68^abc^	39.50 ± 1.04^a^
MCV (fL)	58.48 ± 3.17^a^	56.37 ± 4.48^a^	51.94 ± 1.02^a^	55.62 ± 1.91^a^	53.20 ± 1.22^a^	55.88 ± 1.27^a^
MCH (Pg)	23.73 ± 0.71^a^	21.09 ± 0.74^ab^	20.12 ± 0.47^b^	19.48 ± 0.79^b^	19.83 ± 0.46^b^	22.23 ± 0.51^a^
MCHC (g/dL)	39.44 ± 1.64^ab^	36.94 ± 0.85^ab^	38.84 ± 0.56^ab^	35.28 ± 1.878^b^	37.35 ± 0.25^ab^	39.87 ± 0.36^a^
Platelet/Cmm	342.00 ± 66.00^a^	219.00 ± 34.00^a^	183.00 ± 10.00^a^	590.00 ± 90.00^b^	314.00 ± 114.00 ^a^	371.00 ± 103.00 ^a^
WBCs/Cmm	14.32 ± 2.57^ab^	27.95 ± 4.38^b^	10.34 ± 3.02^a^	26.56 ± 5.16^b^	16.85 ± 1.39^ab^	9.23 ± 1.33^a^
Calcium (mg/dL)	9.82 ± 0.31^a^	8.99 ± 0.19^a^	9.52 ± 0.14^a^	9.57 ± 0.63^a^	9.60 ± 0.09^a^	9.82 ± 0.29^a^
Phosphorus (mg/dL)	7.51 ± 0.09^a^	7.78 ± 0.31^a^	7.72 ± 0.22^a^	8.08 ± 0.37^a^	7.52 ± 0.15^a^	7.61 ± 0.092^a^
Sodium (mEq/L)	150.67 ± 2.02^a^	144.75 ± 2.54^a^	145.40 ± 2.80^a^	147.50 ± 3.37^a^	152.25 ± 1.49^a^	153.60 ± 2.50^a^
Potassium (mM)	7.97 ± 1.47^a^	7.14 ± 0.32^a^	7.71 ± 0.09^a^	6.60 ± 0.33^a^	7.69 ± 0.16^a^	7.26 ± 0.37^a^

*Hb* hemoglobin, *RBCs* red blood cells, *HCT* hematocrit "volume of RBCs in blood", *MCV* mean corpuscular volume "average volume of RBCs", *MCH* mean corpuscular Hb "average mass of Hb/RBCs", *MCHC* mean corpuscular Hb concentration "concentration of Hb in a given volume of RBCs", *WBCs* white blood cells. Results are expressed as mean ± SE (n = 5). Different letters are significantly different for the same row at P < 0.05.

### The protective effects of BVJ alone and with DMSA on rat neurotoxicity induced by Pb

#### Reduction of Pb level and the improvement of rat body weight

**Figure 2:**
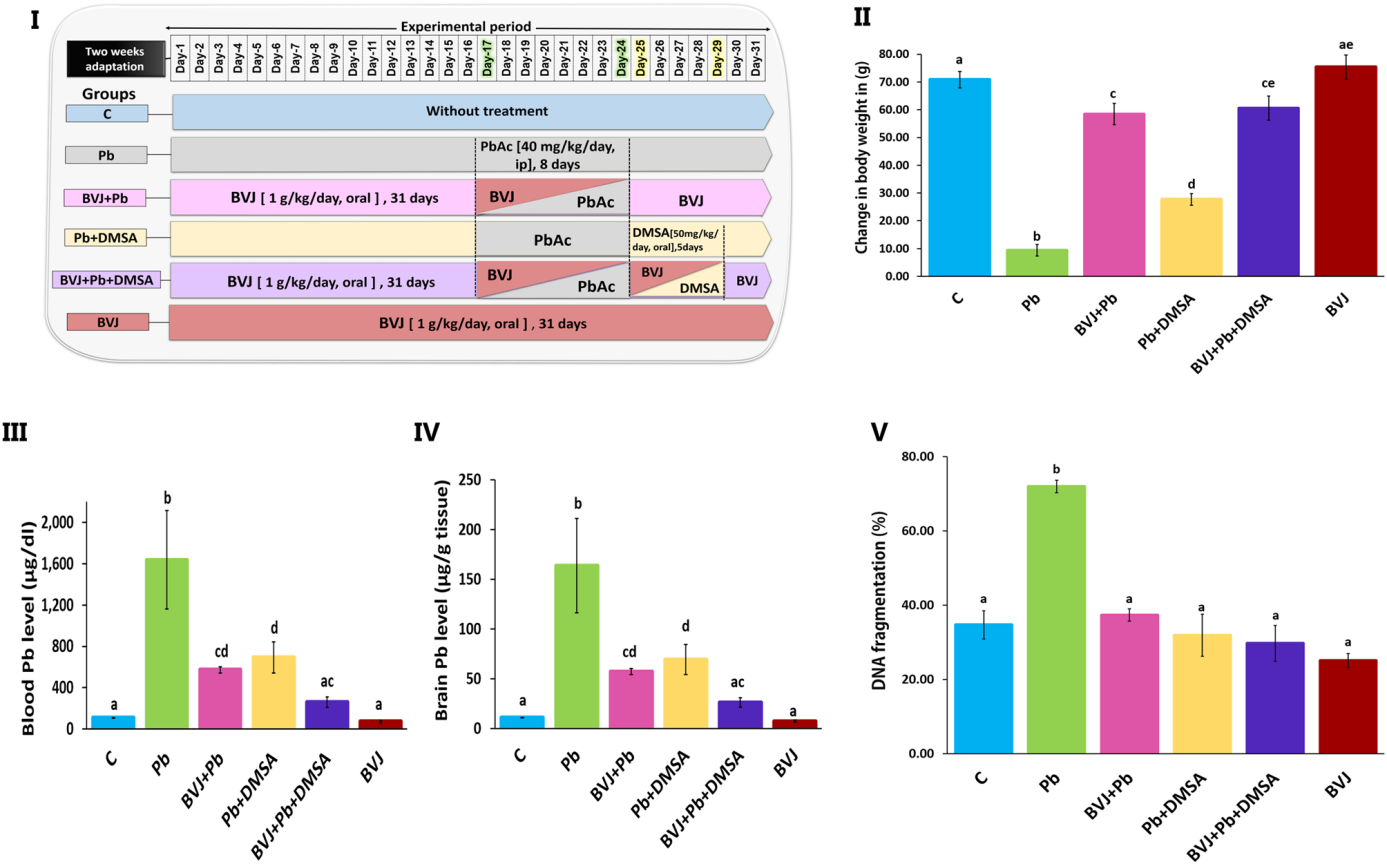
The ameliorating effects of *Beta vulgaris* juice (BVJ), dimercaptosuccinic acid (DMSA), and their combination (BVJ + DMSA) on the blood and brain Pb and DNA fragmentation in Pb-intoxicated rats. Where (**I**): An illustration of the current experimental design, (**II**): the change in the body weight of rats in all the studied groups, (**III**): the blood in all the studied groups, (**IV**): the brain Pb levels in all the studied groups, and (**V**) the % of DNA fragmentation level in all the studied groups. The results are shown as mean ± SE (n = 8). Different letters for the same parameter are significantly different at *p* < 0.05.*C* control,; *ip* intraperitoneal.

As shown in Fig. 2III, IV, the injection of rats with Pb acetate (Pb group) resulted in a significant (*p *˂ 0.05) elevation of Pb levels in the blood and brain compared to the control group. In addition, the change in the body weight of rats after 31 days was greatly reduced than the control (Fig. 2II). Administration of BVJ before, during, and after the injection of Pb acetate (BVJ + Pb group) obviously reduced Pb levels in both the blood and brain. Similarly, treatment with DMSA after Pb injection (Pb + DMSA group) decreased Pb levels in both the blood and brain. Likewise, the change in body weight was significantly (*p *˂ 0.05) improved in rats of (BVJ + Pb) group more than rats in (Pb + DMSA) group as compared to the Pb group. Treatment of rats with BVJ and DMSA together (BVJ + Pb + DMSA group) revealed a highly synergistic depleting effect (CI ˂ 1) of the Pb level in blood and brain (Table 3). Also, these treatments caused the synergistic elevation in the body weight change as compared with Pb group. Otherwise, the administration of BVJ alone to healthy rats caused non-significant changes in either the Pb level or the body weight.

**Table 3:** The Combination index (CI)^#^ values for the studied parameters on the mixture of *Beta vulgaris* juice (BVJ) and meso-2,3-dimercaptosuccinic acid (DMSA).

Effect	CI	Parameters
**In vitro antioxidant models**		
Synergistic	0.430 ± 0.000	DPPH (mg/mL)
Synergistic	0.477 ± 0.134	NO (mg/mL)
Antagonistic	31.578 ± 7.523	Hydroxyl radical (mg/mL)
Synergistic	0.397 ± 0.272	Superoxide radical (mg/mL)
**Change in body weight**		
Synergistic	0.412 ± 0.041	Body weight (g)
**Lead level**		
Synergistic	0.037 ± 0.012	Blood lead level (µg/dL)
Synergistic	0.090 ± 0.030	Brain lead level (µg/g tissue)
**Serum electrolytes**		
Synergistic	0.856 ± 0.036	Phosphorus (mg/dL)
Synergistic	0.941 ± 0.027	Sodium (mEq/L)
Synergistic	0.975 ± 0.017	Calcium (mg/dL)
Synergistic	0.795 ± 0.087	Potassium (mmol/L)
**Oxidative stress parameters**		
Additive	1.000 ± 0.037	TAC (µg BHT Eq/g tissue)
Synergistic	0.429 ± 0.044	Lipid peroxidation (pmol/g tissue)
Synergistic	0.563 ± 0.062	GSH (mg/g tissue)
Synergistic	0.860 ± 0.054	SOD (IU/mg protein)
Synergistic	0.820 ± 0.078	GPX (IU/mg protein)
**Inflammatory parameters**		
Synergistic	0.382 ± 0.077	IL-6 (pg/g tissue)
Synergistic	0.715 ± 0.067	NO (mmol/g tissue)
**Neurotransmitters-associated enzymes**		
Synergistic	0.563 ± 0.161	MAO-A (ng/mg protein)
Synergistic	0.477 ± 0.047	AChE (pmol/mg protein)
**DNA fragmentation**		
Synergistic	0.876 ± 0.234	DNA fragmentation (%)

^#^CI value of < 1 shows synergistic effect; > 1 shows antagonistic effect; = 1 shows additive effect.

*TAC* total antioxidant capacity,* DPPH *α, α-diphenyl-β-picrylhydrazyl,* NO* nitric oxide,* GSH* Reduced Glutathione, *SOD* superoxide dismutase,* GPX* glutathione peroxidase,* IL-6* Interleukin-6, *MAO-A* monoamine oxidase-A, *AChE* acetylcholine esterase.

#### Reduction of DNA fragmentation (DNAF) induced by Pb toxicity

The current findings revealed that Pb injection increased significantly (p ˂ 0.05) the DNAF level in rat brain tissue (Fig. 2V). However, the treatments with BVJ, DMSA, or their combination after Pb injection, significantly (p ˂ 0.05) reduced DNAF induced by Pb as compared with the Pb group. Where, the DNAF level became near to the control level in case of BVJ treatment, while it became lower than the control level after treatment with DMSA, or (BVJ and DMSA). The CI study revealed synergistic (CI ˂ 1) protective effect of the investigated combination of BVJ and DMSA against the elevation in DNAF induced by Pb toxicity (Table 3). BVJ administration to healthy rats caused decreased DNAF level as compared to the control group.

#### Improvement the changes in hematological parameters and serum electrolytes induced by Pb toxicity

The results disclosed that Pb administration affects the blood picture by decreasing the levels of hemoglobin (Hb), red blood cells (RBCs), and hematocrit (HCT) as well as elevating white blood cells (WBCs) (Table 2). Treatment with BVJ before, during, and after Pb injection improved the blood picture which changed by Pb toxicity. In contrast, treatment with DMSA after Pb injection was unable to improve the blood picture as in BVJ treatment.

Hence, the levels of Hb, RBCs, HCT, and mean corpuscular Hb concentration (MCHC) were significantly (*p *˂ 0.05) decreased, while the levels of platelets and WBCs were significantly (*p *˂ 0.05) increased. On the other hand, treatment with BVJ and DMSA together after Pb injection improved the blood criteria. The results also revealed that BVJ administration to the healthy rats did not change the blood picture (Table 2). Likewise, the results demonstrated that there were non-significant changes in the electrolyte concentrations in all studied groups (Table 2).

#### BVJ reduced the OS induced by Pb in rat brain

**Figure 3:**
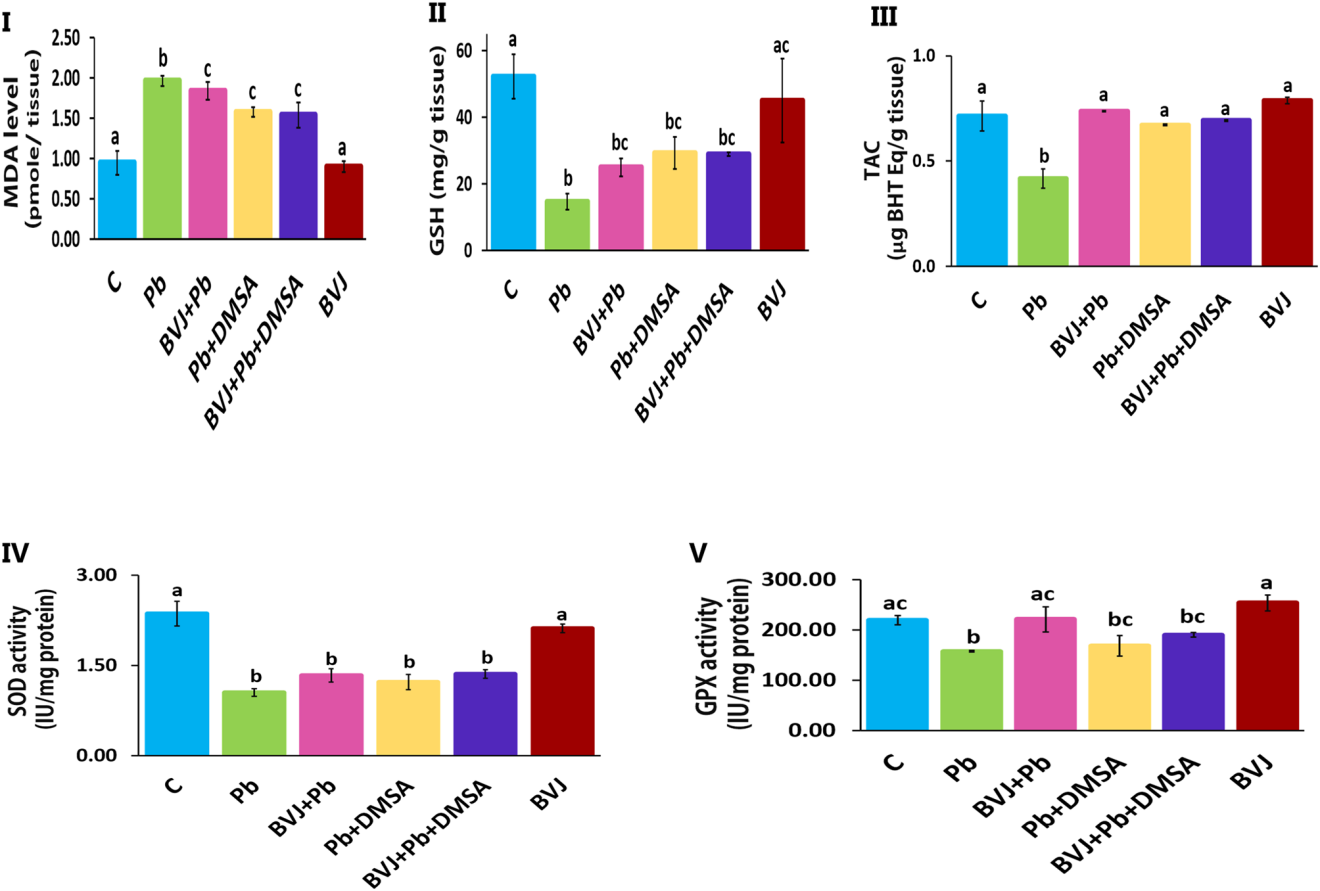
The alleviating influences of *Beta vulgaris *juice (BVJ), dimercaptosuccinic acid (DMSA), and their combination (BVJ + DMSA) on Pb-induced oxidative stress in the brain tissue of Pb-intoxicated rats. Where (**I**): lipid peroxidation level in the brain tissue of all the studied groups, (**II**): reduced glutathione (GSH) level in the brain tissue of all the studied groups, (**III**): total antioxidant capacity (TAC) in the brain tissue of all the studied groups, (**IV**): the activity of superoxide dismutase (SOD) in the brain tissue of all the studied groups, and (**V**) glutathione peroxidase (GPX) in the brain tissue of all the studied groups. The results are shown as mean ± SE (n = 8). Different letters for the same parameter are significantly different at *p* < 0.05.*C* control.

The injection of Pb into rats dramatically increased the level of lipid peroxidation (Fig. 3I) and decreased the level of GSH and total antioxidant capacity (TAC) (Fig. 3II, III, respectively), and the activity of SOD and GPx (Fig. 3IV, V, respectively). Treatment with BVJ before, during, and after Pb injection showed significant (*p *˂ 0.05) suppression in lipid peroxidation level and substantial elevation as well as normalization of TAC level and GPx activity relative to the Pb group. Also, this treatment increased GSH level (significantly) (*p *˂ 0.05) and SOD activity (non-significantly) as compared to the Pb group. While the administration of DMSA after Pb injection was significantly (*p *˂ 0.05) lowered the lipid peroxidation induced by Pb in brain tissue, but its level was still higher than the control. Additionally, this treatment improved significantly the TAC and GSH levels and GPx activity and also, SOD activity (non-significantly) as compared to the Pb group where some parameters became near to their controls.

Similarly, treatment with BVJ and DMSA together influenced on the antioxidant indices. Regarding the CI value, the combination of BVJ and DMSA had synergistic (CI ˂ 1) action on the OS parameters, except for TAC that showed an additive (CI = 1) effect (Table 3). On the other hand, the healthy rats administered with BVJ alone for 31 days reported no significant changes in the OS indices in the brain tissue.

#### BVJ reduced the inflammation induced by Pb in rat brain

**Figure 4:**
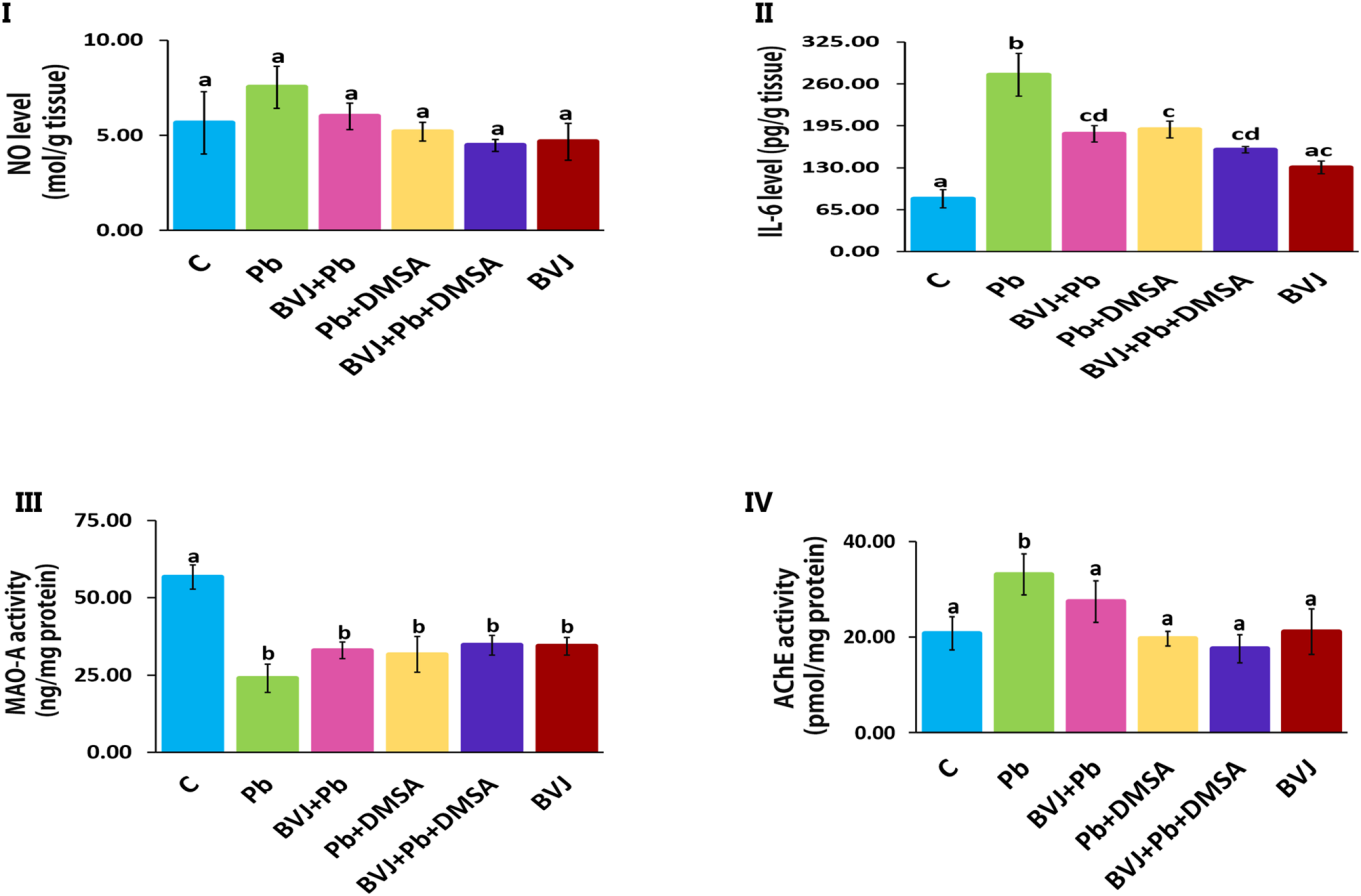
The alleviating effects of *Beta vulgaris* juice (BVJ), dimercaptosuccinic acid (DMSA), and their combination (BVJ + DMSA) on Pb-induced inflammation and disturbance to the neurotransmitters-associated enzymes in brain tissue of Pb-intoxicated rats. Where (**I**): nitric oxide (NO) level in the brain tissue of all the studied groups, (**II**): interleukin (IL)-6 level in the brain tissue of all the studied groups, (**III**): the activity of monoamine oxidase (MAO)-A in the brain tissue of all the studied groups, and (**IV**): the activity of acetylcholine esterase (AChE) in the brain tissue of all the studied groups. The results are shown as mean ± SE (n = 8). Different letters for the same parameter are significantly different at *p* < 0.05.* C* control.

The findings reported that Pb injection did not influence on the NO level of brain tissue (Fig. 4I). Likewise, treatment with either, (BVJ and DMSA together) or DMSA after Pb injection showed no significant changes in the brain NO level. Also, administration of healthy rats with BVJ after Pb injection appeared no significant change in the brain NO level.

Otherwise, the interleukin (IL)-6 level was significantly (p ˂ 0.05) elevated in rat brain tissues which injected with Pb (Fig. 4II). Treatment with BVJ, (BVJ and DMSA together), or DMSA after Pb injection dramatically inhibited the elevation in IL-6 comparable to the Pb group, but the level stilled higher than the control. The CI analysis demonstrated the synergistic (CI ˂ 1) effects of the combination between BVJ and DMSA on depleting the levels of both NO and IL-6 in the brain tissue (Table 3). Further, the administration of BVJ to healthy rats caused a slight elevation in IL-6 level as compared with the control group.

#### BVJ improved the disturbance in the activities of monoamine oxidase-A and acetylcholine esterase in rat brain

The results showed that Pb intoxication caused a drastic depletion of the monoamine oxidase A (MAO-A) activity with a significant elevation in acetylcholine esterase (AchE) activity as compared to the control (Fig. 4III, IV). However, the treatments with BVJ, (BVJ and DMSA together) or DMSA did not significantly protect the depletion of MAO-A activity comparable to the Pb group. In contrast, these treatments significantly prevented the unusual increase of AChE activity by Pb and preserved its activity close to the control. The CI values of treatment with BVJ and DMSA together for the activities of MAO-A and AChE, were less than one (Table 3). Therefore, BVJ displayed a synergistic protective effect on both enzymes. Also, the intake of BVJ alone to the healthy rats was able to dramatically decrease the activity of MAO-A, but did not influence on AChE activity as compared to the control.

### Histopathological results

**Figure 5:**
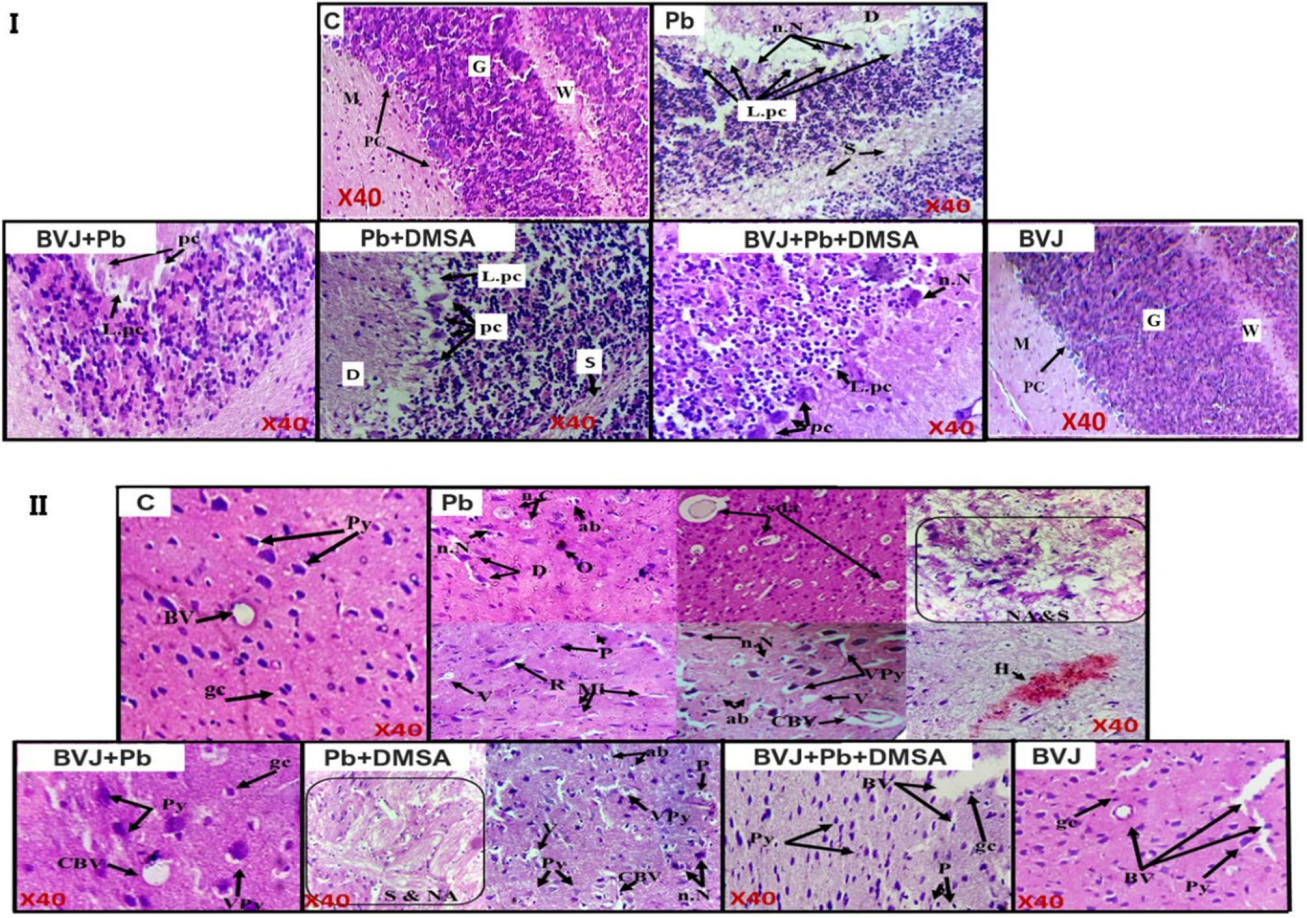
Representative photomicrographs of the cerebellum and the cerebral cortex in the brain of control (C) and Pb-intoxicated rats with and without administration of *Beta vulgaris* juice (BVJ), dimercaptosuccinic acid (DMSA), or their combination (BVJ + DMSA). Where (**I**): photomicrographs of the cerebellum, and (**II**): photomicrographs of the cerebral cortex. *ab* apoptotic bodies, *BV* blood vessel, *CBV* congested blood vessel, *D* degeneration, *G* glial layer, *gc* glial cells, *H* hemorrhage, *L.pc *loss of Purkinje cells, *M* molecular layer, *Mi* microgliosis, *NA* necrotic area, *n. C* neuronal chromatolysis, *pc* Purkinje cells, *W* white matter layer, *n.N* neuronal necrosis, *O* osseous metaplasia, *P* pyknosis, *Py* pyramidal cells, *R* reactive gliosis, *S* spongiosis, *sda* swollen degenerate axons, *V* vacuolization,* VPy* vacuolized pyramidal cells, × *40* magnification of the image.

**Figure 6:**
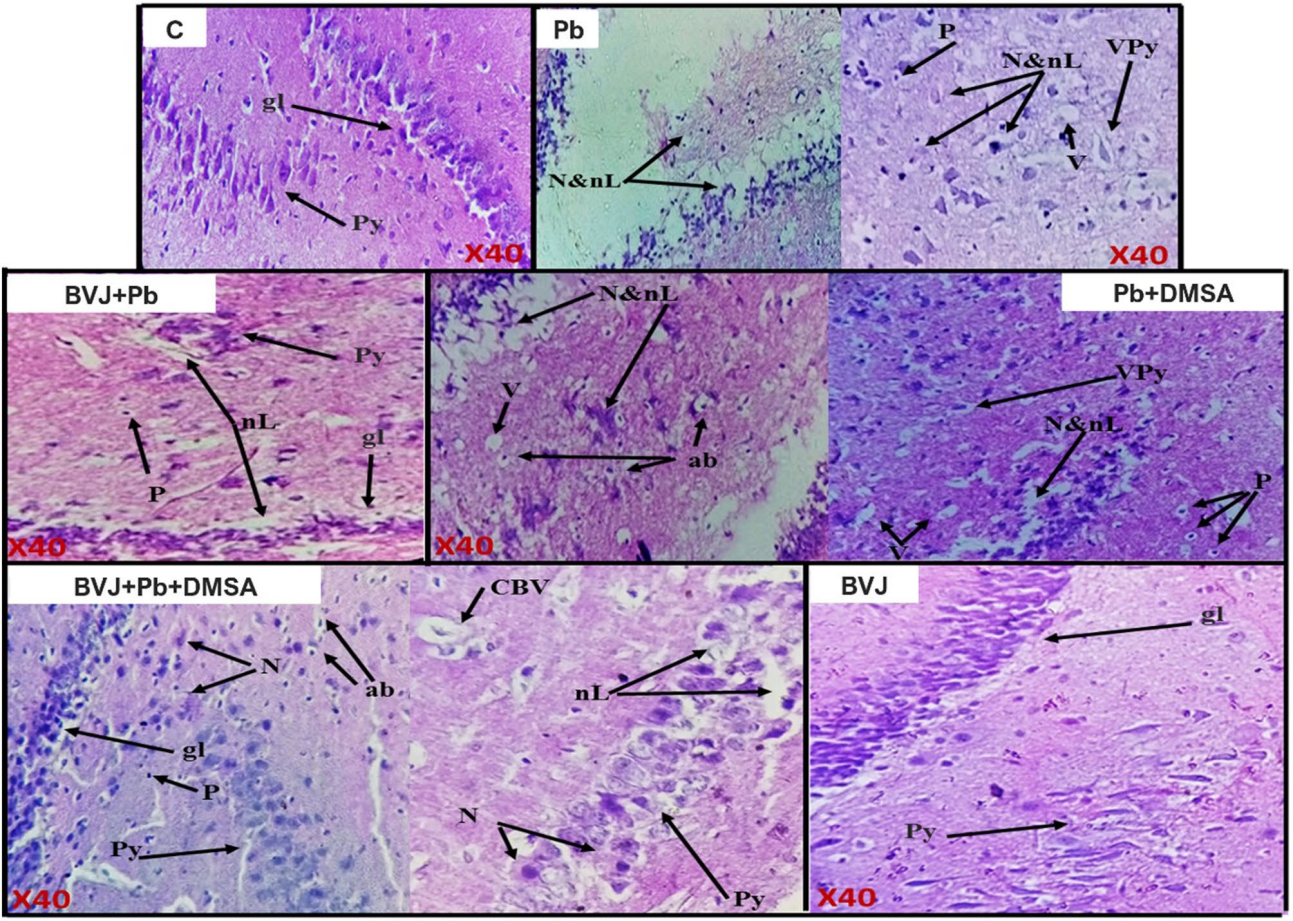
Representative photomicrographs of the hippocampus in the brain of control (C) and Pb-intoxicated rats with and without administration of *Beta vulgaris* juice (BVJ), dimercaptosuccinic acid (DMSA), or their combination (BVJ + DMSA). *ab* apoptotic bodies, *CBV* congested blood vessel,* gl* granular layer,* N* necrosis,* nL* neuronal loss,* P* pyknosis,* Py* pyramidal cells,* V* vacuolization, *VPy* vacuolized pyramidal cells, × *40* magnification of the image.

In this study three areas of the brain were chosen for histopathological study, cerebellum (Fig. 5I), cerebral cortex (Fig. 5II), and hippocampus (Fig. 6). The brain sections taken from the control rats (C group) displayed normal cerebellum, cerebral cortex, and hippocampus structures. The light photomicrographs showed the proper cerebellar architecture with three cortical layers "granular, Purkinje, and molecular" and a white matter layer. The cerebral cortex revealed typical pyramidal nerve cells, glial cells, and blood vessels. Similarly, the hippocampus showed normal pyramidal nerve cells.

The microscopic examination of the brain sections of rats injected with Pb (Pb group) has detected severe damage in the three brain regions. The cerebellum showed serious destruction of Purkinje cells, neuronal necrosis, serious molecular degeneration, as well as serious white matter spongiosis with vacuolization. Multiple alterations were detected in the cerebral cortex such as vacuolization of pyramidal cells, congestion of blood vessels, and focal reactive gliosis. Moreover, degenerated neurons, microgliosis, neuronal necrosis, swollen of some degenerate axons, pyknosis of glial cells, severe spongiosis with vacuolation, and with many other disturbances were detected in this brain area. The hippocampus revealed extreme necrosis and neuronal loss with pyramidal cell vacuolation, serious vacuolation, and neuronal pyknosis.

Otherwise, treatment with BVJ before, during and after Pb injection (BVJ + Pb group) maintained normal cerebellar architecture, except for a small loss of Purkinje cells. The cerebral cortex showed few vacuolated pyramidal nerve cells and congested blood vessels with normal glial cells, while the hippocampus disclosed few neuronal loss and neuronal pyknosis.

Also, the results showed that the treatment with DMSA after Pb injection (Pb + DMSA group) restored most of Pb toxicity in the three investigated brain areas. In the cerebellum, only moderate disturbance in Purkinje cells, molecular layer and white matter were noted. The microscopic observation of the cerebral cortex showed moderate vacuolation of pyramidal neurons, congestion of the blood vessels, neuronal necrosis, apoptotic bodies, and pyknosis of glial cells with other damage. The hippocampus demonstrated moderate necrosis, neuronal loss with vacuolization of pyramidal nerve cells, and neuronal pyknosis.

The administration of the combination of BVJ and DMSA together (BVJ + Pb + DMSA group) after Pb injection reported a slight loss of Purkinje cells with mild neuronal necrosis in the cerebellar sections. However, there were few vacuolated pyramidal nerve cells with normal glial cells and blood vessels in the cerebral cortex. Moreover, the results observed normal glial cells, few vacuolated pyramidal cells with mild necrosis, some neuronal pyknosis, and mild congestion of blood vessels in the hippocampus.

Otherwise, the administration of BVJ alone for healthy rats for 31 days showed normal cerebellar architecture with normal cortical layers and white matter. Also, it observed normal cerebral cortex and hippocampus architectures in the brain sections of these rats.

### Results summary

**Figure 7:**
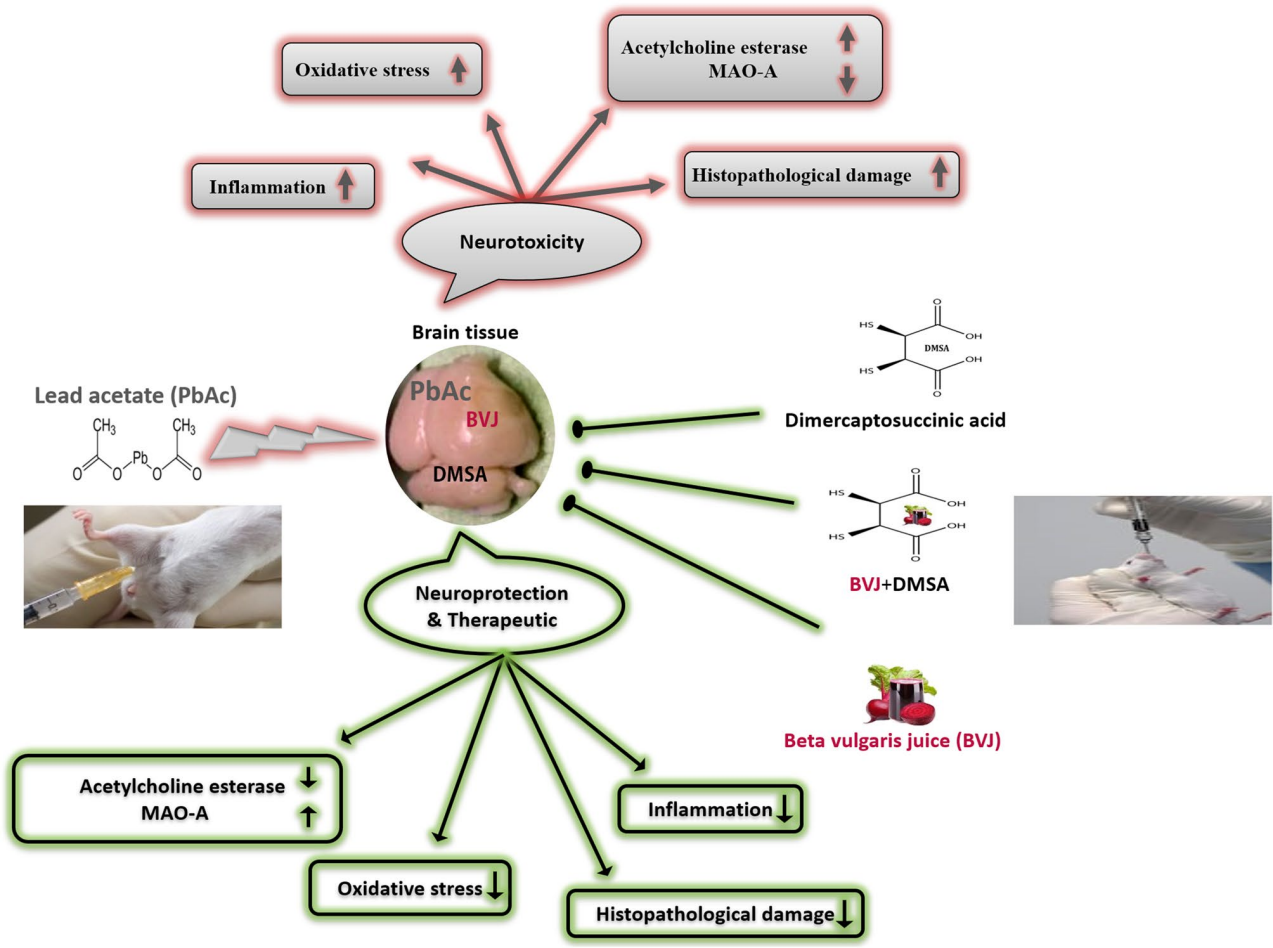
Graphical abstract.

All results obtained in this study were summarized in Fig. 7.

## Discussion

The brain is the most critical organ for lead toxicity^9^. Both of the PNS and the CNS are affected by lead toxicity^19^ but, the mechanisms of lead neurotoxicity are complex and still not clearly understood^17,32^. However, in adults, the PNS is more affected, while in children, the CNS is more affected^33^. It is well identified that Pb-induced neuronal injury is correlated with oxidative challenge, neuroinflammation, and the enhancement of programmed cell death, which may play an important role in the development of the neurodegenerative diseases. The Pb capability to substitute for the calcium ions, allowing its passage across the blood–brain barrier and its accumulation in the brain via calcium-ATPase pumps^19^. Where the increasing in Pb intake may lead to the reduction of calcium steady state^34^. Consequently, both the peripheral and central nervous systems are disrupted^34^. This substitution may results in many neurological disorders including mental retardation, brain damage, behavioral problems, and may lead to several diseases like Alzheimer, Parkinson, and Schizophrenia^35^.

The histopathological results of this study (Figs. 5, 6) revealed extreme damage in the cerebellum, cerebral cortex, and hippocampus parts of the brain, which confirm the serious damage induced by Pb in the brain^36^. The histopathological results are in harmony with the biochemical outcomes. Where, the data showed a significant elevation in MDA level with significant reduction in GSH level and the activities of SOD and GPx in in rat brain homogenate after Pb administration when compared to the control (Fig. 3). This indicates that lead induced OS in brain tissues, therefore, Pb administration led in turn to a drastic decrease in TAC of brain tissue (Fig. 3). OS is a balance loss of ROS production and elimination in tissues and cellular components. In the cells, MDA is one of the eventual products of peroxidation of polyunsaturated fatty acids. A rise in the free radicals, such as O_2_^–**۰**^, and hydrogen peroxide (H_2_O_2_) radicals, causes the overproduction of MDA^37^ MDA is well recognized as an indicator of OS and antioxidant status and an elevation in its level is a substantial index of lipid peroxidation^37^. In the body, SOD initiates the defense action versus O_2_^–**۰**^ radicals since it is believed as the most effective antioxidant. GPx provides a second way of protection against organic hydroperoxides and inorganic (and ROOH and H_2_O_2_ respectively). Where GPx, in the incidence of GSH, stimulates the reduction of these hydroperoxides. GSH works as a nucleophilic scavenger of many compounds through chemical and enzymatic mechanisms. In this study, the depletion of GSH may be related to the increased demand of GSH for lipid hydroperoxide metabolism to terminate free radical reactions. While GSH depletion could contribute to the activation of lipid peroxidation^37^. The peroxidation of lipid contents of the membrane leads to a lack of cell perfection, an increment in membrane permeability, and alteration of Ca^2+^ homeostasis that donate to cell death. In addition, the lipid peroxidation caused the destruction of mitochondrial through the formation of the permeability transition pore, and priming activation of apoptosis^38^. Therefore, our results revealed an increase in DNAF after Pb administration (Fig. 2V). The fundamental mechanism underlying Pb-induced oxidative damage to membranes is linked with changes in its fatty acid composition^39^. Where Pb induces the elongation of arachidonic acid which might be accountable for the increased lipid peroxidation^39^. The second mechanism for Pb-induced OS is the inhibition of the antioxidant defense systems of cells. Pb and other metals such as Cd and Hg have a high affinity for sulfhydryl (SH) groups. Mercaptides are created with the SH group of cysteine, which are less stable complexes^8^. So, Pb alters the antioxidant activities by inhibiting functional SH groups in several enzymes such as δ-aminolevulinic acid dehydrase, SOD, catalase, and GPx. In addition, GPx, CAT, and SOD are possible targets for lead toxicity because these enzymes depend on different essential trace elements for proper molecular structure and activity^39^. Inhibition the δ-aminolevulinic acid dehydrase^40^ leading to the accumulation of δ-aminolevulinic acid. δ-aminolevulinic acid resembles and competes with γ-aminobutyric acid, a neurotransmitter in the hypothalamus, cortex, and other nervous system tissues. This can stimulate γ-aminobutyric acid receptors and induce neurotoxicity^8^ through generating H_2_O_2_ and O_2_^•−^ radicals as well as binding to oxyHb producing OH^•−^ radicals^41^ resulting in the induction of the OS^41^. 4,5-dioxovaleric acid, (the final oxidation product of δ-aminolevulinic acid) is an effective alkylating agent and play an important role in DNA damage^39^. Moreover, lead accumulation in tissues has reported inducing oxidative DNA damage, through strand break^38^. Moreover, other studies suggested that Pb induces alteration in gene expression^35^ and it appears to react with zinc-binding sites on an important DNA-linked protein, human protamine^35^.

Additionally, the present results showed that Pb administration caused significant reductions in the rat body weight and the levels of Hb, RBCs, and HCT with a significant increase in WBCs as compared the control (Table 2). However, the levels of minerals were non-significantly changed after Pb administration (Table 2). The reduction in Hb may be related to inhibition of its biosynthesis through the inhibition of δ-aminolevulinic acid synthase, δ-ALA dehydratase and ferrochelatase by Pb^40^. Additionally, Pb can bind with RBCs and inhibits pyruvate kinase "keeps cellular energy homeostasis"^42^ and pyrimidine 5′-nucleotidase (essential for RBCs maturation)^43^. The inhibition of these enzymes destabilizes the RBCs membrane and decreases its fluidity leading to a shortage of the erythrocyte life span, hemolytic anemia, and ROS generation^42,43^. High levels of ROS lead to oxidation of the cellular biomolecules, especially, the biomembrane lipids causing lipid peroxidation^44^. These results are agreed with the previous studies^18,26,32,36,45^.

Inflammation is considered as a beneficial process under normal condition via controlling the innate response to biological or physical triggers such as trauma, infection and other pathogens that may disrupt homeostasis and cause diseases^46^. Cytokines include interleukins, chemokines and other signalling molecules which can be classified into pro and anti-inflammatory mediators^46,47^. These mediators are working together in balance to perform the overall effect of the physiologically inflammatory response for repairing cells and tissue injuries. Nevertheless, the disturbance of cytokines' balance leading to inappropriate activation of inflammatory processes that can cause excessive cell and tissue damage ultimately leading to many pathological conditions^46^. Our results revealed that Pb administration caused a significant elevation in the level of interleukin-6 (IL-6) in the brain tissues compared to the control group (Fig. 4II). This may be due to increasing its expression in response to Pb as suggested by the previous studies^48^. IL-6 is one of the potent pro-inflammatory cytokines that recruit neutrophil influx and induce prostaglandin synthesis, activation, and proliferation of immune cells. This may have a dangerous effect on the differentiation and growth of neurons^49^.

MAO is an intracellular flavin-containing enzyme that catalyzes the oxidative deamination of monoamine neurotransmitters like norepinephrine and epinephrine in the brain and peripheral tissues^50^. MAO is localized on the outer membrane of mitochondria regulating the extracellular concentration of monoamines^50^. In addition, AChE is the key enzyme in the nervous system responsible for the degradation of acetylcholine “an important neurotansmitor” during neurotransmission, which is necessary for learning and memory^51^. Lead toxicity is known to decrease MAO^52^ and AChE activities in the crude homogenate of the rat brain^5,53^. It is thought that the inhibition of AChE and MAO activities under the effect of Pb is caused by the binding of metals to the functional groups of proteins like imidazole, sulfhydryl, and carboxyl^8^. Our results exhibited that Pb administration significantly decreased MAO-A activity but significantly increased AChE activity. The activation of AChE activity by Pb toxicity is in agreement with Ghareep et al. (2010) and others^54–56^. Till now the causes of the activation of AChE by Pb are not known. It may be due to the effect of Pb on the gene expression of this enzyme. So many studies must be carried out in this field. The previous studies revealed that Pb inhibits MAO activity and this inhibition is dose and its duration dependent. The change in MAO activity leads to various mental and neurodegenerative disorders^52^. Also, activation of AchE may lead to alteration in the exploratory behavior and locomotor activity of rats^54^.

However, it is known that Pb intoxication can be effectively treated using chelation therapies such as DMSA. Previous studies showed that DMSA has some drawbacks including, CNS convulsions, essential metal loss, hepatotoxicity, nephrotoxicity, headache, nausea, hypertension, respiratory arrest, skin rashes, thrombocytopenia, and neutropenia^25,26^. Therefore, in the present study, the protective role of BVJ on rat neurotoxicity induced by Pb was investigated and the results were compared with those resulted from the treatment with DMSA. Besides, the possible synergistic antineurotoxicity action of the combination between BVJ and DMSA was determined. Additionally, the phytochemical components of BVJ and its characterization were identified to be used in the discussion of the results of the biological role of BVJ against Pb-induced brain toxicity.

The present results showed that BVJ contains various phytochemical constituents with different concentrations as shown in (Table 1). Where, the phenolics, betacyanins, betalains, betaxanthins, flavonoids, and flavonols are present in a large amount. Additionally, HPLC analysis revealed the presence of many phenolic acids (Table 1). Since, Myricetin, Quercetin, Naringenin, Pyrogallol, Caffeic acid and Chlorogenic acid are present in great amounts (Table 1). Also BVJ contains carotenoids and vitamins^31^, especially vitamin C^26^. All these compounds have antioxidant activities and protective role for cellular components against OS and can improve the clinical outcomes for several diseases^31^. Owed to these constituents, BVJ observed antiradical activity against DPPH, NO, O^-^·, and OH^‾·^ radicals as shown in Fig. 1. The present results were in harmony with some previous studies^26,57^. Also, BVJ contains minerals such as iron and zinc^31^. It has been demonstrated that dietary supplement or foods rich in calcium, zinc, magnesium, and phosphate may decrease the absorption of Pb^58^.

Likewise, the current data showed that DMSA has antioxidant efficiency against the tested free radicals (Fig. 1) and this may be related to its structure that contains –SH groups "dithiol compound"^59^. DMSA revealed the higher potency than BVJ toward DPPH and OH^‾·^ radicals, while BVJ showed better affect against NO radical. Moreover, the results showed that the combination between DMSA and BVJ had more efficacy (synergistic action, CI ˂ 1) than that of DMSA or BVJ in a separate form, except for the OH^-**۰**^ radical (Table 3). In general, these results demonstrated that the combination between DMSA and BVJ will increase the therapeutic potential against Pb toxicity.

On the other hand, the present results revealed that the level of Pb in blood and brain was significantly reduced in rats treated with BVJ before, during, and after Pb administration as compared to the Pb group, and thus leading to the normalization of the blood parameters (Table 2). This indicates that the active gradients of BVJ such as polyphenols, phenolic acids and flavonoids^60^, and betalains^26,61^ may act as metal chelators^1^. These results agree with previous studies which showed that BVJ prevented silver pathological effects and restored renal functions levels to the control values via interacting with silver ions facilitating their clearance^62^. Besides it showed hepatoprotective activity aganit pb toxicity by reducing blood and liver Pb level and reducing the OS^26^. Also, the activity of AChE was not being elevated, but the activity of MAO-A was non-significantly elevated relative to the Pb group (Fig. 4III, IV). The efficiency of BVJ as a chelator and its effect on the neurotransmitter enzymes was similar to DMSA that used here as a standard chelator for Pb. However, DMSA revealed a slight improvement on the blood parameters and no effect on the tested minerals (Table 2).

Furthermore, the current results elucidated an attenuation of brain damage induced by Pb in rats treated with BVJ before, during and after Pb administration as shown from the results of brain histopathology. Where, slight damage appears in the morphology of the three studied regions of the brain in rats treated with BVJ before, during and after Pb administration. In addition, BVJ treatment decreased lipid peroxidation and apoptosis induced by Pb since the levels of MDA, NO, and DNAF were decreased with an elevation in GSH level, and the activities of GPx, and SOD as compared with the Pb group. This indicates that BVJ has antioxidant, radical-scavenging properties and anti-apoptotic activity which reduce the programmed cell death. This conclusion was confirmed by our results which showed that the total antioxidant activity of BVJ is very high. The antioxidant and antiapoptotic effects of BVJ are related to the effects of its bioactive ingredients which are mentioned before (Table 1).

Several studies showed that the antioxidative characteristics of plant polyphenols may arise from their reactivity as an electron or hydrogen donors, their ability to stabilize unpaired electrons, and their ability to end Fenton reactions^63^. In general, the polyphenolic compounds can prevent oxidative damage as a result of their ability to scavenge ROS. Also, they improve GSH level and the activities of SOD and GPx^64^. Moreover, treatment with BVJ before, during and after Pb administration diminished the inflammation in brain tissues induced by Pb, as obvious from the reduction of IL-6 level as compared with the Pb group (Fig. 4II). This indicates that BVJ has anti-inflammatory activity which may be due to the effect of its bioactive compounds (Table 1). These results agree with the previous studies which showed that BVJ has anti-inflammatory activity^20,61^.

Otherwise, the results showed that treatment with DMSA after Pb intoxication reduced the Pb level in brain tissues and blood. Also, the results showed that DMSA significantly reduced lipid peroxidation level and DNAF induced by Pb toxicity and restored the TAC of the brain tissue, but had no effect on the level of GSH and the activity of GPX and SOD (Fig. 3). This indicates that thiol groups in DMSA play an important role as chelating agent for removing Pb besides its role as antioxidant. These results are in accordance with the previous findings of Ercal et al.^59^. In addition, treatment with DMSA reduced the IL-6 level as compared with Pb group. Also, after this treatment the histopathological findings showing moderate damage in the morphology of the three studied regions of the brain (Figs. 5, 6).

In general, the present study not only proved the protective effect of BVJ against Pb toxicity, but also the safety of its administration for healthy rats for 31 days on rat's brain tissue. This was confirmed by the biochemical and histopathological outcomes. Only, the activity of MAO-A was significantly decreased as compared to the control. This may be owed to the presence of quercetin (Table 1; Fig. 1I) orits derivativesin BVJ constituents. These flavonoid compounds can act as MAO inhibitors and used in the therapy of depression, anxiety, and neurodegenerative disorders^65^.

On the other hand, we also evaluated the protective effect of the combination between BVJ and DMSA against Pb-induced neurotoxicity. From the value of CI (Table 3), this combination exhibited synergistic action (CI ˂ 1) towards all the studied parameters except for the TAC which showed additive effect (CI = 1). As previously published, DMSA is an effective Pb chelator, but various side effects are associated with its therapy^66^. In contrast, BVJ is safe and effective in protecting from Pb toxicity the combination of both BVJ and DMSA enhanced their efficacy leading to the improvement of DMSA therapy. Therefore, this study suggested the use of BVJ with DMSA during the treatment of lead poisoning, while BVJ can be used alone for protection.

In summary, our findings clearly revealed that BVJ has a vigorous efficiency in the protection from Pb-induced toxicity in brain tissue by reducing blood and brain Pb and preventing oxidative and inflammatory stress. The combination of both BVJ and DMSA revealed synergistic antioxidant and anti-inflammatory, antiradical and chelating potentials against neurotoxicity induced by Pb. Therefore, BVJ is a promising extract in protection from Pb toxicity and its combination with DMSA exerted a potent therapeutic effect for this damage.

## Materials and methods

### Chemicals

Gallic acid, catechin, quercetin, ursolic acid, Butylated hydroxytoluene, DMSA, thiobarbituricacid (TBA), Folin–Ciocalteau reagent, reduced GSH, DPPH, 2,2′-azino-bis(3-ethylbenzothiazoline-6-sulphonic acid) (ABTS) and bovine serum albumin (BSA) were purchased from Sigma-Aldrich (St. Louis, MO, USA). Lead acetate was obtained from ISO-CHEM, France. Potassium, sodium, and phosphorous colorimetric kits were obtained from Spectrum, Egyptian Company for Biotechnology (S.A.E), Egypt. Calcium colorimetric kit was purchased from Biosystems (Barcelona, Spain). Rat interleukin (IL)-6 ELISA kit was obtained from Ray Biotech (Norcross, USA). The MAO-A ELISA kit was supplied from FIVE photon Biochemicals (California, USA). Amplite colorimetric AchE assay ELISA kit was purchased from AAT Bioquest, Inc. (California, US). Other chemicals were obtained with a high grade.

### Juice preparation

The BV plant roots (NCBI: txid 3555) were obtained from the Local market in Alexandria, Egypt, during September, then the roots were washed and peeled. The BVJ was prepared using a household dry juice extractor then the extract was dried using the Lyophilizer (Telstar, Terrassa, Spain) to obtain the powdered juice (BVJ, yield 13 g/100 mL BVJ), which stored at − 20 °C until used.

### Quantification of BVJ phytochemicals content

Some of the phytochemical compounds in BVJ were determined, including betalains, phenolics (flavonoids and flavonols), and triterpenoids. Total betalains in BVJ was determined as the sum of betacyanins and betaxanthins concentrations according to the method of Stintzing et al.^67^. The diluted solution (1:3 wt/v) of BVJ was measured at three different wavelengths, 536 nm, 485 nm, and 650 nm (impurities), then the concentrations of betacyanins or betaxanthins were calculated using the following formula:

$$\left[ {{\text{betacyanins }}\;{\text{or }}\;{\text{betaxanthins}}\; \, ({\text{mg/L}}) \, = \frac{A \times DF \times M.W \times 1000}{{\epsilon\times i}}} \right],$$

where, A = A_536_–A_650_ (for betacyanins) or A_485_–A_650_ (for betaxanthins), DF: dilution factor, M.W: molecular weight of betacyanins or betaxanthins (550 and 336 g/mol, respectively), $\epsilon$: molar extinction coefficient for betacyanins or betaxanthins (60,000 and 48,000 L/mol cm, respectively), i: path length (cm).

Total phenolics in mg equivalents of GA/g BVJ were quantified using Folin–Ciocalteu reagent, which was reduced by BVJ phenols producing a blue color solution with a maximum absorbance at 750 nm^68^. Total flavonoids (mg catechin equivalents/g BVJ) were measured using 10% AlCl_3_ and 5% NaNO_2_ solutions^69^ While total flavonols (mg QR equivalents/g BVJ) were estimated using 2% AlCl_3_ and 50 g/L sodium acetate solutions^70^. The triterpenoids (µg UA equivalents/g BVJ) were determined colorimetrically using vanillin color reaction^71^.

### Investigation of the phytochemical compositions of BVJ using HPLC

Phytochemical components of BVJ were identified and quantified by agilent 1260 infinity HPLC series (Agilent Technologies, Palo Alto, CA, USA) as indicated previously with slight modification^72^. Briefly, 20 µL of BVJ was separated on a Kinetex EVO C18 column (100 mm × 4.6 mm) using a ternary linear elution gradient. The elution was performed using 0.2% H_3_PO_4_, methanol, and acetonitrile and the detection was done at 284 nm. Pure standards were run in the same chromatographic conditions to match the retention items.

### In vitro antioxidant activities of BVJ, DMSA and their combination

The antioxidant activities of BVJ, DMSA, and their combination (BVJ + DMSA, 20:1) were evaluated using various methods, including, DPPH, NO, O_2_^−**۰**^and OH^‾·^radicals scavenging activities. The antioxidant activities were compared between BVJ, DMSA, and (BVJ + DMSA) using Asc as a standard antioxidant. The IC_50_ value (50% inhibitory concentration) for each radical was determined using the % of inhibition values [(A_Control_ − A_Sample_/A_Control_) × 100] at different studied samples (BVJ), (DMSA), (BVJ + DMSA) or (Asc) concentrations^72^.

Different concentrations (0.062–2 mg/mL) of each studied sample and Asc were prepared for using in each experiment. DPPH free radical scavenging activity was determined by incubating the DPPH radicals with the studied samples or Asc, separately for 30 min then the absorbance of the non-scavenged radical was read at 490 nm^73^. NO scavenging activity was measured using the Griess reaction to produce a bright-reddish-purple colored azo dye following the method of Marcocci et al.^74^. In this reaction, sodium nitroprusside and Griess reagent (1% sulfanilamide, 2% phosphoric acid and 0.1% naphthylethylenediamine dihydrochloride) were used. The O_2_^−**۰ **^scavenging activity was measured as described previously^75^ in the presence of pyrogallol and the absorbance of the reaction mixture was measured at 320 nm every 30 s for 5 min. OH^‾·^ radicals cavenging activity was measured at 510 nm using salicylic acid assay^76^.

### Animals and treatments

All animal experiments in this study were carried out in correspondence with the Alexandria University Guidelines for Care and Use of Laboratory Animals. The institutional animal care and use committee of Alexandria University (AlexU-IACUC) approved the protocol of this study (AlexU-IACUC protocol no. AU 04 20 05 16 3 02).

Seventy-two male four week-old Albino rats and weighing (90–100 g) were obtained from Theodor Bilharz Research Institute, Giza, Egypt. Animals were kept in stainless steel wire bottom cages at about 30 °C with a 12 h light–dark cycle and allowed free access to a standard commercial diet and tap water. Rats were acclimatized under these conventional conditions for 2 weeks, and then they were randomly classified into six groups (twelve rats in each) as illustrated in Fig. 2I. The PbAc dose (40 mg/kg body weight "bw") was chosen and given intraperitoneally (ip)according to previous study^77^. The BVJ dose (“1 g BVJ dissolved in distilled water/kg bw = 8 mL juice/kg bw, where 1 g of BVJ contains 17.26 mg galic acid equivalent as total phenolic compounds) was chosen and given orally using oral cavage according to the previous study^78^. Also, the dose of DMSA “50 mg/kg bw” was chosen and given orally according to the previous studies^79^. The C group: control rats without any treatment; Pb group: rats were (ip) injected with lead acetate (PbAc) in a dose of [40 mg/kg body weight (bw)/mL distilled deionized water "dd.H_2_O"], for a consecutive 8 days; (BVJ + Pb) group: rats were administered with BVJ before, during and after Pb injection, where rats were administered orally (using oral cavage) with BVJ [1 g/kg bw/8 mL dd.H2O], daily for 31 days (the experiment period), while PbAc (as in Pb group) was given for 8 days from 17^th^–24^th^ day; (Pb + DMSA) group: rats were firstly injected with PbAc as described above then treated with chelating drug DMSA (50 mg/kg bw/mL dd.H_2_O), for a consecutive 5 days; (BVJ + Pb + DMSA) group: rats were treated BVJ and DMSA in combination, where rats were administered with BVJ before, during and after PbAc injection (i.e. for 31 days as described before in BVJ + Pb), also the rats were treated with DMSA for 5 days as in mentioned before; and the BVJ group: rats were administered with BVJ only with the same dose mentioned before for 31 days. At the end of the experimental period (day 31), the animals were sacrificed under carbon dioxide euthanasia following the euthanasia guidelines in the Guide for the Care and Use of Laboratory Animals. Then, the blood was collected by cardiac puncture and brain tissues were removed immediately. The heparinized blood was used in the examination of the complete blood count (CBC) and blood Pb level. In addition, serum samples were obtained by centrifugation of the non-heparinized blood for 15 min at 6000 rpm for the electrolytes quantification. The brain tissues were washed with cold saline solution (0.9% NaCl) and small portions were fixed in 10% formalin for histopathological examination. The remaining brain tissue was kept at − 80 °C until used in the biochemical analyses.

### Analysis of hematological indices, Pb level and electrolytes

The hematological indices of rats in each studied group were assessed using CBC Analyzer (Nihon Kohden, Celttac, Japan). The Pb concentrations in both blood and brain samples were determined using atomic absorption spectrometry (Varian, model spectr AA 240, Mulgrave, Australia) after their digestion with HNO_3_/H_2_O_2_^80^_._ While the electrolytes calcium, phosphorus, sodium and potassium were quantified using the specific kits.

### Determination of DNAF

The level of DNAF was determined spectrophotometrically as indicated previously with modifications^81^. In brief, 25 mg of brain tissue was homogenized in phosphate buffer 1 M, pH 7.0 then 250 µL of DNA lysis buffer "TTE" (1 M Tris-HCI pH 8, 0.2% Triton X-100, and 0.5 M EDTA), and 10 µL proteinase K were added and vortexed. Then, the homogenate was centrifuged at 15,000 rpm (4 °C) for 10 min. Where, 0.5 mL of TTE solution was added to the pellets and 50 µL ice-cold NaCl (5 M) was mixed vigorously with the supernatant before DNA precipitation using isopropanol. The DNA was washed with ethanol (70%), dissolved in deionized water-RNase solution, and finally incubated for two days at 37 °C. The absorbance (A) was recorded using a nanodrop spectrophotometer at 260 nm, then DNA fragmentation (%) was measured according to the formula: [A_supernatant_/(A_supernatant_ + A_pellet_)] × 100.

### Determination of OS markers

The level of MDA (the most abundant aldehyde in lipid peroxidation) and the antioxidant markers including GSH level, and the activities of SOD and GPx besides total antioxidant capacity were assessed in brain homogenate to evaluate the oxidative stress. The brain tissue from each studied group was homogenized in 5% TCA, 3 mM EDTA for GSH analysis and in phosphate buffer (0.1 M, pH 7.0) for analysis of the remaining parameters. The MDA level was measured colorimetrically as thiobarbituric acid reactive substances "TBARS"^82^. This method depends on the reaction between MDA with TBA and measure the resulting pink chromogen colour at 532 nm. The GSH was determined spectrophotometrically using 5,5′-dithiobis-(2-nitrobenzoic acid)^83^. This chromogen was reduced by the sulfhydryl group (-SH) of GSH yielding a yellow-colored product that was recorded at 412 nm. The GPx activity was determined colorimetrically using cumene hydroperoxide and GSH as substrates^84^. The activity of Cu/Zn SOD was assessed via spontaneous auto-oxidation of pyrogallol and the change in absorbance during 2 min was measured at 420 nm^85^. The unit of enzyme activity is expressed to the amount of enzyme which suppresses 50% of the auto-oxidation rate of 20 mM pyrogallol under the standard conditions. Total protein content in the brain homogenate samples was quantified by the Lowery method using BSA as a standard^86^ for calculating the specific activity (unit activity/mg of protein) of the antioxidant enzymes. The TAC of the brain tissue homogenates in each studied group was determined by the ABTS^+^ radical cation-decolorization assay^87^. The reduction of the ABTS^+^ radical to ABTS by the antioxidants in the homogenate samples was detected by the disappearance of the blue-green color and recorded at 734 nm.ABTS^+^ radical was prepared before mixing with the tissue homogenates by incubating 7 mM ABTS in the dark for 16 h with 140 mM potassium persulfate at 25 °C. The % of ABTS^+^ radical inhibition was calculated and used in the quantification of the total antioxidant activity as µmol BHT equivalent/g brain using the BHT calibration curve.

### Quantification of the inflammatory mediators

The levels of both NO and IL-6 were measured in the brain tissues of all studied groups. The NO level was determined colorimetrically using Griess reagent '0.1% naphthylethylenediamine dihydrochloride, 1% sulfanilamide, and 2% phosphoric acid"^88^. The intensity of the produced bright-reddish-purple azo dye was measured at 490 nm. IL-6 was determined at the protein level using a specific ELISA kit following the manual protocol.

### Assessment of the neurotransmitters-associated enzymes

The activities of the MAO-A and AChE were quantified in the brain tissues of all the experimental groups. The protein levels of both enzymes were determined using the specific ELISA kits following the manufacturer's protocol.

### Evaluating the potency of BVJ and DMSA combination

The combination of food extracts and drugs may give higher (synergistic), lower (antagonistic), or no new (additive) effect compared to the effect of the drug alone. Evaluation of this novel influence can be achieved via the determination of the CI value. This value was calculated by dividing the expected value on the observed value. The expected value between compound 1 and compound 2 for the in vitro antioxidant methods was calculated by the addition of the half values of each compound together. While this value is determined for the in vivo studied parameters as [(observed value for compound 1)/(control value)] × [(observed value for compound 2)/(control value)] × (control value)^89^. The CI value may be ˂ 1 (synergistic effect), or equal to 1 (additive effect), or ˃ 1 (antagonistic effect) ^90^.

Calculation of CI

$${\text{CI}} = \frac{{{\text{Expected }}\;{\text{value }}\;{\text{(EV) }}}}{{{\text{Observed}}\;{\text{ value}}\;{\text{ (OV)}}\;{\text{ of }}\;{\text{the }}\;{\text{combined}}}}$$

For in vitro antioxidant methods

$${\text{EV}} = \raise.5 ex\hbox{$\scriptstyle 1 $}\kern-.1em/ \kern-.15em\lower.25ex\hbox{$\scriptstyle 2 $} {\text{ BVJ}}_{{{\text{OV}}}} + \raise.5 ex\hbox{$\scriptstyle 1$}\kern-.1em/ \kern-.15em\lower.25ex\hbox{$\scriptstyle 2$} {\text{ DMSA}}_{{{\text{OV}}}}$$

Where, the Observed value (OV) $\text{of the combined}$: the observed value of (BVJ + Pb + DMSA).

For the in vivo parameters

$$\begin{gathered} {\text{EV}} = \, \left[ {\left( {{\text{BVJ}} + {\text{Pb}}} \right)_{{{\text{OV}}}} /{\text{control }}\;{\text{value}}} \right] \, \times \, \left[ {\left( {{\text{Pb}} + {\text{DMSA}}} \right)_{{{\text{OV}}}} /{\text{control }}\;{\text{value}}} \right] \, \times {\text{ control}}\;{\text{ value}} \hfill \\ {\text{OV: }}\;{\text{The}}\;{\text{ observed}}\;{\text{ value }}\;{\text{of }}\left( {{\text{BVJ}} + {\text{Pb}} + {\text{DMSA}}} \right). \hfill \\ \end{gathered}$$

### Histopathological study

The histopathology was done by the routine protocol for three regions in the brain, cerebellum, cerebral cortex, and hippocampus. After fixation of the brain specimens, they were embedded in paraffin wax and cut into thin slices with a thickness of 5 µm with a microtome and stained with hematoxylin and eosin^32^.

### Statistical analysis

The data were expressed as mean ± standard error (SE). The statistical analysis was performed by using one-way analysis of variance (ANOVA). The post hoc analysis (Dunnett’s test) using SPSS (Statistical Package for Social Sciences) software version 25 was followed. The statistical difference values were considered at *p* < 0.05. Heat map diagrams were obtained by Clust Vis web tool "https://biit.cs.ut.ee/clustvis/"^91^.

## Abbreviations

AchE: Acetylcholinesterase
Asc: Ascorbic acid
BV: *Beta vulgaris*
BSA: Bovine serum albumin
BHT: Butylated hydroxytoluene
*BVJ*: BV juice
CI: Combination index
CBC: Complete blood count
DPPH: α,α-Diphenyl-β-picrylhydrazyl
GA: Gallic acid
GSH: Glutathione
GPX: Glutathione peroxidase
HCT: Hematocrit
*Hb*: Hemoglobin
HPLC: High-performance liquid chromatography
*IL-6*: Interleukin-6
MDA: Malondialdehyde
MCHC: Mean corpuscular Hb concentration
DMSA: Meso-2,3-dimercaptosuccinic acid
MAO-A: Monoamine oxidase A
NO: Nitric oxide
QR: Quercetin
ROS: Reactive oxygen species
RBCs: Red blood cells
SOD: Superoxide dismutase
TBA: Thiobarbituric acid
TBARS: Thiobarbituric acid reactive substances
TAC: Total antioxidant capacity
UA: Ursolic acid
